# Vaulting mechanics successfully predict decrease in walk–run transition speed with incline

**DOI:** 10.1098/rsbl.2012.1121

**Published:** 2013-04-23

**Authors:** Tatjana Y. Hubel, James R. Usherwood

**Affiliations:** Structure and Motion Laboratory, Royal Veterinary College, University of London, Hatfield, Hertfordshire AL9 7TA, UK

**Keywords:** walk, run, transition, incline, inverted pendulum

## Abstract

There is an ongoing debate about the reasons underlying gait transition in terrestrial locomotion. In bipedal locomotion, the ‘compass gait’, a reductionist model of inverted pendulum walking, predicts the boundaries of speed and step length within which walking is feasible. The stance of the compass gait is energetically optimal—at walking speeds—owing to the absence of leg compression/extension; completely stiff limbs perform no work during the vaulting phase. Here, we extend theoretical compass gait vaulting to include inclines, and find good agreement with previous observations of changes in walk–run transition speed (approx. 1% per 1% incline). We measured step length and frequency for humans walking either on the level or up a 9.8 per cent incline and report preferred walk–run, walk–compliant-walk and maximum walk–run transition speeds. While the measured ‘preferred’ walk–run transition speed lies consistently below the predicted maximum walking speeds, and ‘actual’ maximum walking speeds are clearly above the predicted values, the onset of compliant walking in level as well as incline walking occurs close to the predicted values. These findings support the view that normal human walking is constrained by the physics of vaulting, but preferred absolute walk–run transition speeds may be influenced by additional factors.

## 1. Introduction

Terrestrial locomotion is characterized by distinctive gaits such as walking, trotting and galloping in quadrupeds, or walking and running in bipeds. When and why a change of gait occurs has been the subject of various studies, with conflicting reports regarding the importance of energetic cost and mechanical boundaries as the prevailing factor to induce gait transition [1–7]. Human adults show a discrete switch from walk to run at a transition speed close to a Froude number (*Fr* = *V*^2^/(*gL*), where *V* is mean speed, *g* gravity and *L* leg length) of 0.5 or a relative velocity 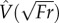 of 0.7 [2,3,5,7,8]. We continue to explore the possibility of a mechanical boundary as gait determiner, following a path first explored by Alexander [9] and later modified by Usherwood [4].

The compass-gait model is based on the inverted pendular motion of the centre of mass (CoM) when vaulting over the stance leg. It is reductionist in its assumption of a point mass representing the body, vaulting over a stiff, massless leg of constant length and without double support [10]. It is effective in accounting for midstance forces in walking humans [11]. Computational analysis shows that under the reductionist conditions, the inverted pendular motion is indeed the most economic way of walking at low speeds [12]. More level ‘compliant’ walking styles are theoretically [12,13] and demonstrably [14,15] more costly than inverted pendular walking, because the legs perform work as they deflect under load during much of stance (time when foot is in contact with the ground).

The maximum speed at which inverted pendular walking is possible is restricted by gravity providing sufficient centripetal force to prevent foot take-off or slipping. Alexander [9] determined the critical speed to be at a Froude number of 1, a prediction later modified by Usherwood [4] under consideration that take-off conditions are most limiting at the extremes of leg angle (early and late stance) owing to the combination of increased centripetal force requirements (as the body moves faster) and a reduced weight component along an angled leg. Limitations to leg angles based on conditions at early and late stance lead to a maximum achievable step length related to speed. Consideration of critical take-off conditions at the extremes of stance and incorporation of step length lead to a reduction of predicted walk speed limit down to a Froude number of around 0.5, close to the walk–run transition not only in humans, but also in a number of different sized birds [16,17]. Exceeding the boundaries of inverted pendular walking requires a switch to either running or a different—usually more compliant—walking strategy.

Here, we expand the compass-gait model of vaulting stance to walking up a slope, and consider whether reported [5] and observed decreases in transition speeds with incline are consistent with the constraints predicted by this exceedingly reductionist model. If this is indeed the case, it would further support the hypothesis of a simple mechanical limitation as being one of the causal determinants of gait selection.

## 2. Material and methods

### (a) Compass gait on incline

In compass-gait walking, gravity provides the centripetal acceleration required to keep the CoM on its arcing trajectory, and this requirement is most extreme at the limits of stance, when the gravity component along the leg is also at a minimum; non-take-off conditions are [4]2.1
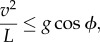
where *v*^2^/*L* is the centripetal acceleration, *v* is instantaneous CoM velocity and *ϕ* leg angle (angle between stance leg and vertical).

**Figure 1.**
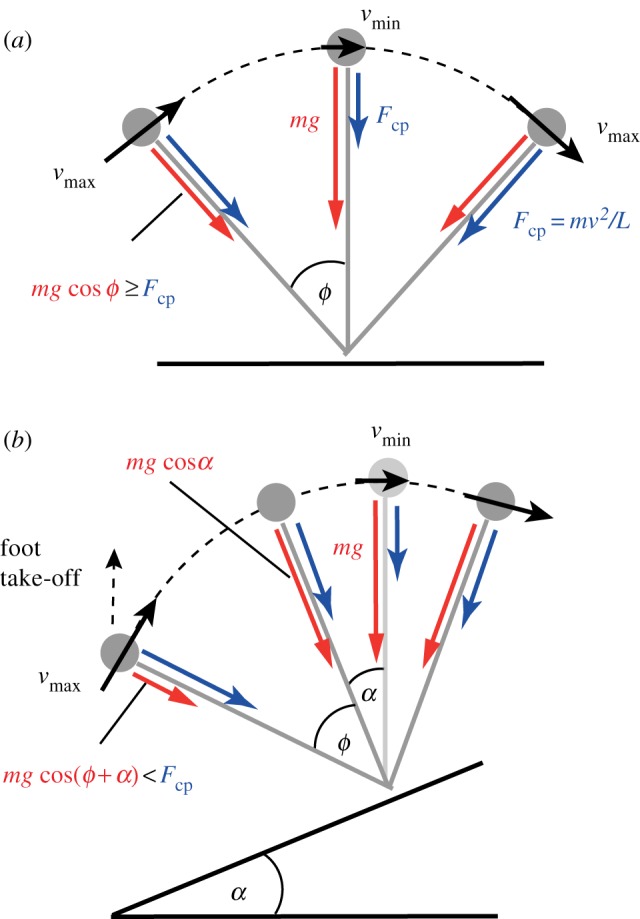
Stance according to the compass-gait model for (*a*) level and (*b*) incline. The CoM (grey circle) vaults over a massless rigid leg of constant length. Centripetal force requirement (*F*_cp*,*_blue), the force necessary to keep the CoM on its circular arc. The component of weight in line with the leg (*mg*, red) decreases with leg angle (*ϕ*). Slope angle (*α*); instantaneous CoM velocity (*v*); mass (*m*) and leg length (*L*). In this example, net speed is the same on level and incline; however, the weight component along the leg is only sufficient to maintain compression throughout stance on the level (*a*), whereas leg tension is required during early stance (thus compass-gait walking is impossible) on the incline (*b*).

The compass-gait model can be extended to incline walking (figure 1). Again assuming that stance follows an inverted pendulum motion, and the leg does not change length during vaulting, leg angles must be symmetrical about the perpendicular to the surface (not gravity). However, maximum step angles still relate to angles from vertical (defined by gravity). Thus, maximum step lengths fall for both incline and decline walking. With similar constraints on step frequency, top walking speeds are, therefore, predicted to decline with greater slope angles. The non-take-off condition becomes2.2
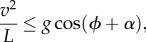
where *α* is the slope angle. Exceeding this condition on an incline is clearly detrimental (see the electronic supplementary materials, figure S1), resulting in greater collision losses, and potentially short and brief steps owing to take-off in early stance. This condition is not expected to constrain decline walking, both because the implications of take-off are not detrimental if they occur late in stance, and the benefit of inverted pendular walking—its economy—may not apply in cases where energy dissipation is actually desirable.

### (b) Experimental set-up

Ten subjects, free of any known pathological disorders, were studied while walking on a treadmill at increasing speed. The subjects ranged in age from 19 to 36 years (28.5 ± 5.8 years, mean ± s.d.), in body mass from 57 to 99 kg (75.25 ± 12 kg), and in leg length (measured as distance from floor to greater trochanter of the femur during standing) from 0.87 to 1.04 m (0.95 ± 0.07 m), with five males and five females. Data were recoded in two sessions. All subjects had previous experience with treadmill walking and running and had performed 2–3 training trials prior to the day of data collection.

The subjects walked on the treadmill on level and incline (slope: 9.8%; 5.6°). The speed of the treadmill was slowly increased over time by accelerating the belt at 0.04 m s^−2^ for 1 s every 2 s (mean acceleration over time: 0.015 m s^−2^ ). The participants were asked to walk at level and incline under two conditions: (i) switching between walking and running at their preferred transition speed and (ii) walking as fast as possible. Five consecutive trials were performed for each condition, with conditions selected in random order. A triaxial accelerometer was positioned at the small of the back of the participants and data sampled at 600 Hz. Treadmill belt speed was simultaneously continuously recorded.

### (c) Analysis

Matlab (MathWorks Inc., MA, USA) was used for data processing. Accelerometer data and speed were filtered using a Butterworth bandpass filter (cut-offs: 1 Hz, 7 Hz). The amplitude of vertical acceleration was used to determine gait transitions. Amplitude increased slightly with walking speed and showed a distinct increase when switching to running (see the electronic supplementary material, figure S2*a*). When walking faster than preferred, the amplitude reached a maximum, then decreased sharply, indicating the switch to compliant-walking; it then increased dramatically at the onset of running (see the electronic supplementary material, figure S2*b*). Peak detection was used to determine an amplitude envelope. A moving average of 15 steps was applied to the amplitude envelope, and the change from positive to negative gradient in amplitude envelope defined the transition to compliant-walking.

Step frequency was calculated using peak detection on horizontal acceleration; vertical acceleration is inappropriate as, unlike horizontal, its relationship with phase of step changes with gait. Step frequency at transition speeds for walking, compliant-walking and running was defined as the mode over the 3 s prior to transition. Step length was calculated by dividing treadmill speed by step frequency and subsequently normalized by leg length. For simplicity, we do not distinguish here between horizontal and belt-based speeds or step lengths as, at an incline of 5.6°, they differ by less than 0.5 per cent. Values were averaged within individuals and subsequently averaged over all participants, and standard errors are displayed based on the number of participants. Statistical significance is determined using ANOVA with participants as random effect conducted in SPSS (v. 20, IBM, NY, USA). Significance levels were determined at 95% (see the electronic supplementary material, tables S1 and S2).

## 3. Results and discussion

**Figure 2.**
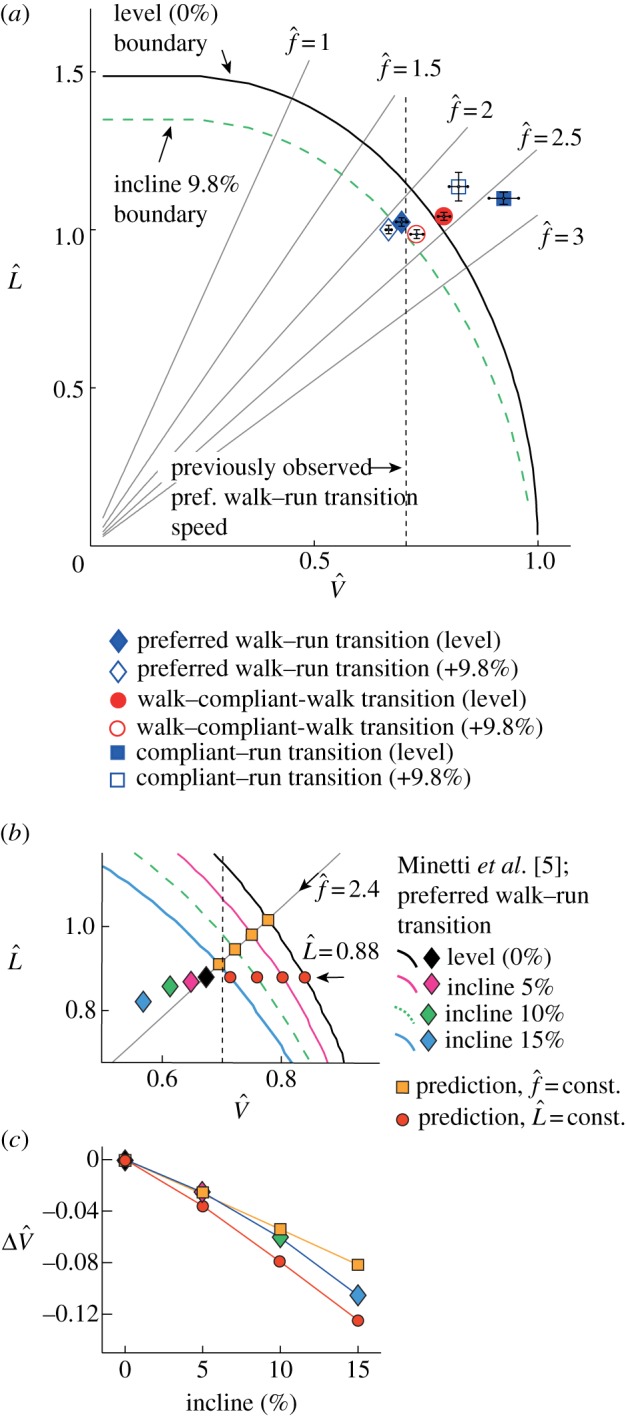
Compass-gait limits of relative velocity and relative step length for level (solid black curve) and inclines (dashed and coloured curves), and various transition speeds. (*a*) Predicted and all observed transition speeds are reduced significantly on the incline. Preferred walk–run transition speeds (diamonds) fall somewhat below the limiting boundaries. Maximum walking speeds exceed the predicted boundaries (squares, *a*), but are only achieved with reduced vertical accelerations indicating compliant-walking. Walk–compliant-walk transition speeds, and their decrease with incline (circles, *a*) are well predicted by compass-gait mechanics. 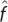: multiples of the passive step frequency assuming a point mass pendular swing-leg of leg length. 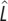: normalized step length (step length/leg length). (*b*) A more extensive previous study [5] shows somewhat lower step length at preferred walk–run transition speed over a range of inclines, but (*c*) the change in relative velocity lies within the prediction if either 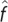 or 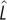 are treated as constant.

The walk–compliant-walk transition indicates the maximum speed people can achieve without deviation from inverted pendular walking (red circles, figure 2*b*). It is remarkably consistent with the boundary predicted by compass-gait mechanics, despite the many deviations of a human from a point mass on a stiff stick-leg. Higher speeds (up to blue squares) are achievable, but are accompanied by a discrete reduction in vertical acceleration amplitude, indicating a transition to more CoM flattened, compliant-walking. This gait is able to exceed the compass-gait speed limit by avoiding the centripetal acceleration requirements through flattening the arc taken by the CoM about the foot during each stance.

The extension of the compass-gait model (equation (2.2)) quantitatively accounts for the observed reduction in walk–compliant-walk transition speed with incline (filled to open red circles, figure 2*a*). Preferred walk–run transition speeds fall well within the compass-gait boundary, perhaps suggesting some form of ‘safety margin’ as the consequences of exceeding the limit—potentially a slip and suddenly brief stance resulting in a trip—may be highly detrimental. That the compass-gait limits, and the extension to incline walking, is informative to preferred walk—run transition speeds is supported by the measurements of Minetti *et al.* [5]. Again, measured transition speeds fall well within the predicted boundaries (figure 2*b*), albeit at somewhat different positions from those reported here, presumably owing to details of methodology. However, *changes* in transition speed are not only consistent with the extended compass-gait boundaries, but are also well and parsimoniously predicted if step frequency is assumed to be constant. With this assumption, using level walking conditions as inputs, preferred transition speed is predicted to fall by approximately 1 per cent per 1 per cent incline (yellow squares, figure 2*b,c*); Minetti *et al.* observed 5.2 per cent over 5 per cent.

To conclude, gait transitions on an incline provide further support that ‘normal’ human walking parameters are constrained by the mechanics of vaulting, and demonstrate that treating walking as an ‘inverted pendulum’ gait has predictive—not merely descriptive—power.
